# The impact of elevation and prediction of climate change on an ultra high‐elevation ectotherm

**DOI:** 10.1002/ece3.70186

**Published:** 2024-09-01

**Authors:** Jie Gao, Zian Wei, Yuanting Jin

**Affiliations:** ^1^ College of Life Sciences China Jiliang University Hangzhou Zhejiang China

**Keywords:** climate change, distribution, elevation, lizard, maximum active time

## Abstract

Climate change may affect the survival and reproduction of ectotherms. The toad‐headed lizard *Phrynocephalus theobaldi*, which holds the distinction of occupying the highest elevation among all reptile species on Earth, with an elevational range from 3600 to 5000 m, represents an ideal model for studying the adaptations to climatic changes across elevational gradients. Here, we used mechanistic and hybrid species distribution models (HSDM) together with characteristic measurements of thermal biology (CT_max_, CT_min_, and T_sel_) to simulate and compare the distribution and activity periods of the lizard across elevations in response to climate change. NicheMapR simulations using only climate factors predicted that all populations will be negatively impacted by climate change (+3°C) by suffering a reduced distribution. However, the impact was clearly reduced in simulations that accounted for thermal physiological traits. Longer activity periods were predicted for all populations during climate change. The suitable distribution is predicted to change slightly, with an increase anticipated for both high and low elevation populations. However, the forecast indicates a more pronounced increase in suitable habitats for populations at higher elevations (>4200 m) compared to those at lower elevations (<4200 m). This study underscores the key influence of climate change on population establishment and stresses the importance of physiological traits in distribution simulation for future studies to understand the potential constraints in animal adaptation to extreme high environments.

## Graphical abstract

Climate change may affect the survival and reproduction of ectotherms. The toad‐headed lizard Phrynocephalus theobaldi, occupying the highest elevation of any reptiles in the earth with an elevational range from 3600 to 5000 m, represents an ideal model for studying the adaptations to climatic changes across elevational gradients. Here, we used a mechanistic model and hybrid species distribution model (HSDM) together with characteristic measurements of thermal biology (CT_max_, CT_min_, and T_sel_) to simulate and compare the distribution and activity periods of the lizard among varied elevations in response to climate change. NicheMapR simulations using only climate factors indicated that all populations can be negatively impacted by climate change (+3°C) because of their decreased estimated distribution. However, the impact clearly reduced in simulations when thermal physiological traits were accounted for, which indicated the great influence of physiology in simulation. Longer activity periods will appear for all populations during climate change. The suitable distribution area could float a little, and the distributing area will enlarge for all higher or lower populations. However, relative higher populations will display their larger, enlarged distributing areas than those of relative lower populations. This study underscores the key influence of climate change on population establishment and stresses the importance of physiological traits in distribution simulation for future studies to understand the potential constraints in animal adaptation to extreme high environments.

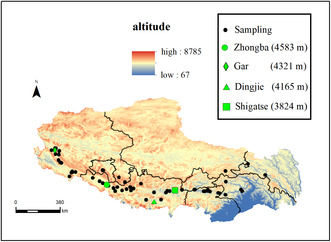

## 1 INTRODUCTION

Climate change represents one of the principal threats to biodiversity (Dukes & Mooney, 1999). It particularly threatens many ecothermic groups, which are less able to adapt quickly to environmental shifts (Mi et al., 2023; Sala et al., 2000; Thomas et al., 2004). Squamate sensitivity to warming differs and impacts vary with altitude (Biber et al., 2023). Understanding how squamates respond to climate warming across altitudes is vital for conservation efforts (Sunday et al., 2014).

Endothermic species maintain their body temperature within an optimal range for normal physiological functions by employing behavioral, physiological, and biochemical thermoregulatory mechanisms in response to environmental thermal variations. In contrast, ectotherms lack the same level of thermoregulatory control; as a result, their survival is impeded by extreme temperatures, whereas moderate temperatures can enhance their physiological function (Kern et al., 2015; Paaijmans et al., 2013; Ruel & Ayres, 1999). Altitudinal change can pose great challenges to the survival of animals (Huey & Kingsolver, 1989). Ectotherms can display physiological (Hou & Huang, 1999; Khatiwada et al., 2020; Zhao et al., 2022), phenotypic (Noble et al., 2018; Trochet et al., 2018), and genetic adaptations (Fu et al., 2007) to variable altitudinal environments. Tropical lizards are predicted to adjust their activity patterns and migratory behavior in response to climate change between 2050 and 2070, with an expected increase in activity periods (Mi et al., 2022). Many mammals and birds also appear to be shifting their migration patterns toward higher latitudes and altitudes in response to climate change (Chen et al., 2011).

Research on ectotherm adaptations to environmental temperatures has predominantly centered on latitudinal responses (Liu et al., 2022; Mi et al., 2022; Weatherhead et al., 2012), focusing less on altitude‐related adaptations (Li et al., 2018). High‐altitude habitats present multifaceted meteorological challenges such as extreme cold, hypoxia, and intense ultraviolet radiation exposure. Investigating squamate behavioral and physiological adaptations to these conditions is crucial to understanding their responses to climate change, which is vital for biodiversity preservation. Consequently, studies addressing squamate's resilience to climatic shifts are of considerable academic and conservation significance.

*Phrynocephalus theobaldi* is widely distributed in the southern Qinghai‐Tibetan Plateau (QTP), southward to the northern slopes of the Himalayas, and north to the Gangdise Mountains and Tanggula Mountains. It is generally found in relatively lower regions of 3594–4200 m in elevation in the east and in relative higher regions over 4200 m, reaching 5054 m, in the alpine desert environment in the west (Jin et al., 2017). We previously discovered that the lizard displayed body size and color variation associated with altitudinal changes (Jin et al., 2017). The individual size of male and female lizards showed nonlinear associations with elevation. Notably, the nonlinear pattern observed in females resembled a U‐shaped curve, with the inflection point appearing at approximately 4200 m (Jin & Liao, 2015). The lower populations (<4200 m) do not have central ventral black spots, but the higher ones (>4200 m) do have these spots, with the size of the black spots increasing with elevation (Jin & Liao, 2015). The black spot on the abdomen of lizards is beneficial to thermal regulation in colder environments of higher altitudes (Jin et al., 2016). It is worthwhile noting that the populations below 4200 m altitude form a distinct lineage, while all other higher altitude populations constitute other three lineages, with these lineages evolving independently (Jin et al., 2017). These results indicated that altitude had an important effect on the phenotypic and genetic adaptation of *P. theobaldi*.

To investigate the performance of *P. theobaldi* in response to climate change in different altitudes, we employed NicheMapR, a software for microclimate and mechanistic niche modeling that has been widely utilized in wildlife research (Enriquez‐Urzelai et al., 2020; Kolbe et al., 2010). NicheMapR integrates a microclimate model using data from geographical locations (Kearney & Porter, 2017) with an ectotherm model (Kearney & Porter, 2020) applicable to a variety of lizard species. The models integrate global monthly climate data, estimating the local hourly surface climate experienced by lizards from the latitude and longitude of the study site, along with terrain attributes, substrate characteristics, and the minimum and maximum levels of shade at each coordinate. Subsequently, the models use the hourly outputs from the microclimate models as inputs for the ectotherm model, which, in conjunction with the lizards' behavior, morphology, and physiological traits, are used to estimate maximal activity windows.

Specifically, we applied an ecological model in conjunction with the thermobiological traits of the *P. theobaldi* population to predict and analyze active duration and habitat suitability. This model aims to explore the following: (1) the impact of future climate change on the overall adaptability of *P. theobaldi* and (2) potential adaptive differences among *P. theobaldi* populations from different elevations in response to global warming. To investigate these questions, it is essential to determine the reptiles' temperature sensitivity (McCann et al., 2014), that is, the select temperature (T_sel_), the critical thermal maximum (CT_max_), the critical thermal minimum (CT_min_), and the factors like habitat suitability (Chiu‐Valderrama et al., 2022) and maximum activity times (Enriquez‐Urzelai et al., 2020). This information will assist in making more accurate predictions about the potential response of reptiles to future climate change (Kolbe et al., 2010).

## 2 METHOD

### 2.1 Sampling

**FIGURE 1:**
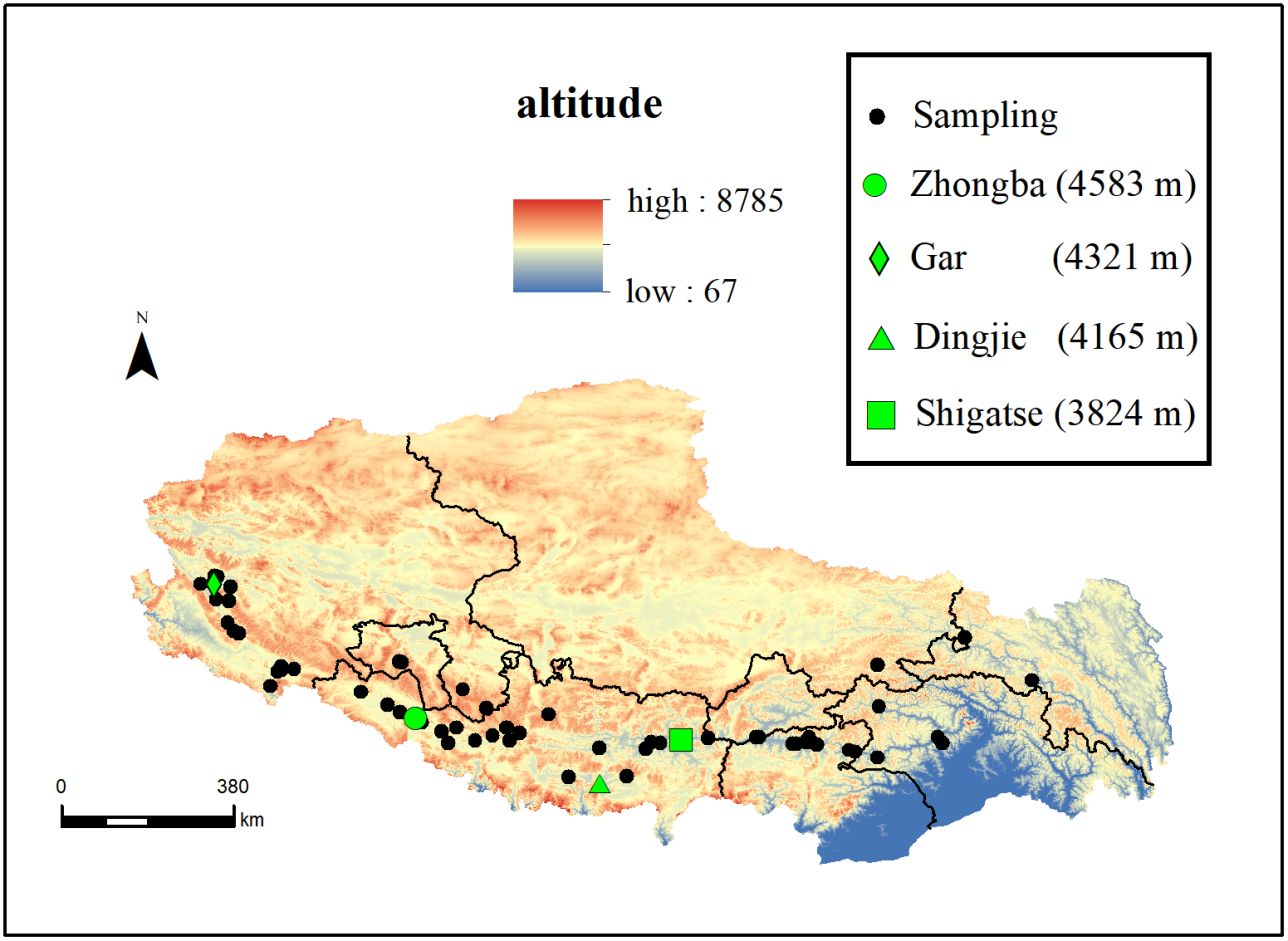
The distribution of *P. theobaldi*. The green diamond, green circle, green triangle and green square indicate the locations of Zhongba (4583 m), Gar (4321 m), Dingjie (4165 m) and Shigatse (3824 m) respectively, and the black dots show the 60 localities at which sightings were made (Jin et al., 2020).

The geographic sampling locations of the lizard obtained from our historical field surveys are shown in Figure 1. Coordinates and elevation data for locations of Zhongba, Gar, Dingjie, and Shigatse are listed in Table 1. The field sampling work was authorized by the Tibet Autonomous Region Forestry Bureau (TARFB). The collection and husbandry protocol for the lizards was approved by the Animal Experimentation Ethics Committee of China Jiliang University.

**TABLE 1:** Elevation, longitude, and latitude of Zhongba, Gar, Dingjie, and Shigatse sites.

Location	Elevation (m)	Longitude (°)	Latitude (°)
Zhongba	4583	84.04 E	29.74 N
Gar	4321	80.04 E	32.40 N
Dingjie	4165	87.70 E	28.46 N
Shigatse	3824	89.33 E	29.32 N

### 2.2 Determination of T_sel_

To determine selected body temperatures (T_sel_) of the *P. theobaldi* from Shigatse, Zhongba, and Dingjie, a total of 45 individuals were chosen for the study—15 individuals were randomly selected from each location. It should be noted that from Shigatse and Zhongba, 7 females and 8 males were selected, respectively, while in Dingjie, 9 females and 6 males were chosen. These selected individuals had a body length ranging from 4.22 to 5.33 cm. Initially, these lizards were acclimatized to a stable ambient temperature of 25°C within a climate‐controlled environment. Subsequently, they were placed into three separate enclosure devices (each measuring 140 cm in length, 30 cm in width, and 40 cm in height) for the experiment. The base of each enclosure was lined with fine sand, which was preheated for 1 h using two 25 W full‐spectrum bulbs to create a thermal gradient ranging from 40.5°C to 26.2°C. This temperature gradient encompasses the potential preferred activity temperature range of the lizard, with the temperature gradient measured from the coolest part of the enclosure surface and air to the hottest point near the heating bulbs. Thermal imaging was conducted at 30‐min intervals within the range of 10:30–11:30 using the Testo 890 infrared thermal imager to measure the dorsal skin temperature of each lizard. This process was repeated three times in 3 days for each individual. Data were recorded to compare the temperature differences among the populations.

### 2.3 Determination of CT_max_

To assess the critical thermal maximum (CT_max_) of *P. theobaldi* from Shigatse, Zhongba, Dingjie, and Gar, a total of 60 individuals (15 random individuals per location) were subjected to a controlled temperature increase. The lizards, initially maintained at an ambient temperature of 25°C, were placed inside perforated 500‐mL plastic bottles to allow for adequate air exchange. These bottles were then transferred to a thermostatically controlled incubator (Wan‐Tian‐Fu‐Kang WTFK‐12L). The temperature within the incubator was gradually elevated at a rate of 0.48°C/min. After reaching 38°C, the bottles were gently rotated every 15 s to ensure even heat distribution. The endpoint of the tolerance test was determined by the loss of righting reflex in the lizards. Immediately after the observed loss of reflex, the lizards were removed from the incubator, and the cloacal temperature was promptly measured using a thermocouple probe.

### 2.4 Determination of CT_min_

In order to ascertain the critical thermal minimum (CT_min_) of *P. theobaldi*, a total of 60 lizards were selected, with 15 individuals from each location: Zhongba, Dingjie, Shigatse, and Gar. Initially, these individuals were acclimated to a uniform room temperature of 25°C. Subsequently, they were placed in 500‐mL plastic bottles equipped with multiple perforations to ensure proper ventilation. The bottles with the lizards were then introduced into an open‐top car refrigerator, which had been pre‐cooled to a target setting of −15°C. This setup served to prechill the environment surrounding the lizards. Following this, the temperature was carefully decrease from the baseline at a rate of 0.5–1°C per min to monitor their thermal tolerance as the temperature approached their critical minimum. The onset of the loss of righting reflex was used as an indicator of low‐temperature tolerance, at which point the cloacal temperature of the lizards was immediately measured using a thermocouple thermometer.

### 2.5 Land surface temperature simulation

We utilized the NicheMapR software integrated with spatial interpolation techniques to simulate soil surface temperature at 1 cm above the ground across Tibet. In the simulations, the midpoint (15th day) of each month was chosen as representative of that month. To simulate the surface temperature for each sample point, we utilized the “micro_global” function within the NicheMapR package, inputting the location coordinates (longitude and latitude), topographic attributes (ground cover, slope, and elevation), and climatic data (daily minimum values of air temperature, wind speed, cloud cover, and relative humidity) (Lin et al., 2019) for a total of 1691 raster grid points, which were obtained by converting the distribution of *P. theobaldi* from the IUCN database using ArcGIS 10.8, and integrating these data with the 60 sampling locations of *P. theobaldi* obtained from field surveys (Jin et al., 2020). This integration process allows for a more accurate representation of the species' distribution. To accurately reflect the terrain's complexity, we generated slope and aspect layers from a Digital Elevation Model (DEM) sourced from the United States Geological Survey, with a resolution of 10 arc min (https://www. usgs.gov). It was assumed that each sample point encompassed an area with vegetation cover conducive to selecting suitable habitat temperatures for *P. theobaldi*. To test this assumption, we utilized Landsat 8–9 OLI/TIRS C2 L2 data to derive the Normalized Difference Vegetation Index (NDVI) and subsequently convert it to Fractional Vegetation Cover (FVC). The remote sensing data were obtained from the Geospatial Data Cloud site, specifically accessible at https://www.gscloud.cn/. Shade was estimated in single patches from FVC. Additionally, we employed all available meteorological station data from the China Meteorological Data Service Center to estimate maximum and minimum air temperatures, leveraging as many stations as possible for increased accuracy.

To explore inter‐annual temperature variations, we modeled surface temperatures at 1 cm under current average conditions and a warmer average scenario (current average +3°C). The model calculates the current average temperature by using the average values of daily minimum and maximum temperatures recorded at each sampling point. The parameters for calculating the average daily low and high temperatures are derived from data provided by the China Meteorological Data Service Center (2010–2020). The 3°C differential used in the warmer average scenario is akin to predicted end‐of‐century warming (2081–2100).

### 2.6 Simulation of maximum active time

Simulations were carried out based on an average‐sized *P. theobaldi* (BM = 5 g) at three different altitudinal zones: low altitude (Shigatse‐3824 m), mid altitude (Dingjie‐4165 m), and high altitude (Zhongba‐4583 m). For each site, simulations were run under two distinct shading scenarios to simplify the analysis. Vegetation was set at unrestricted shading levels (0%–100%) and at restricted levels (50%–100% shading). Under the “low shading level” (0%–100% shade), temperature regulation in *P. theobaldi* was unrestricted, as they could move from fully sunlit to completely shaded conditions, representing a smaller range of shade. In the “high shading level” (50%–100% shade), activity was constrained by more plant cover. This approach is grounded in the principle of parsimony, aiming to balance the complexity and practicality of the model by including only the most critical influencing factors and avoiding excessive parameterization while still capturing the impact of vegetation on microclimate. To assess the potential impact of using uniform shadow parameters across different altitudes, we calculated the mean vegetation shadow coverage rates for each site: Shigatse, Dingjie, and Zhongba, and evaluated the correlation between these rates and the respective altitudes of the locations. We utilized the “ectotherm” function from the NicheMapR software package, employing physiological data collected at three different altitudes and simulated surface temperatures under two shading scenarios, to estimate the maximum activity time of *P. theobaldi* at three geographical locations (see Tables S1 and S3 for more details).

### 2.7 Adaptive zone simulation

HSDM for the lizard included annual activity times forecast by NicheMapR, thermal biology tolerance thresholds of the lizard, and bioclimatic variables situated within the coordinates of the geographical boundaries as defined by the IUCN. We acquired 19 bioclimatic variables from Worldclim v2.1 (Fick & Hijmans, 2017), representing both recent (1970–2000) and future (2081–2100) climate conditions, all at a resolution of 10 arc‐min. In the assessment of model accuracy for predicting the distribution of *P. theobaldi*, 30% of raster grid points were allocated as verification data for testing, while the remaining 70% were used for training purposes. Assessment metrics were Area Under the Curve (AUC) (Merow et al., 2013) and True Skill Statistics (TSS) (Liang et al., 2018). The computed outcomes indicate a commendable performance of *P. theobaldi* model (AUC_Training_ = 0.997, AUC_Test_ = 0.919, TSS_Training_ = 0.762, TSS_Test_ = 0.759). To reduce multicollinearity, we screened variables involved in the model operation using the “usdm” package (Naimi et al., 2014) and their percentage contribution rates. Upon detecting collinearity between bioclimatic variables and ecophysiological responses, we chose to retain the ecophysiological data, as it can reveal the species' ecophysiological adaptive responses to climate change. In the prognostic model for *P. theobaldi*, we ultimately selected the following variables based on their contribution rates: Max Temperature of Warmest Month (BIO5, 37.4%), Activity Period Throughout the Year (24.6%), Isothermality (BIO3, 14.4%), Precipitation of Driest Month (BIO14, 9.3%), Mean Temperature of Driest Quarter (BIO9, 7.7%), Annual Mean Temperature (BIO1, 5.4%), and Thermal Safety Margin (TSM, 1.3%). The percentage values enclosed in parentheses represent the contribution rates of these variable factors within the HSDM. Due to changes in physiological characteristics and bioclimatic variables, suitable habitats are geographical either lost or gained. We calculated the changes in the area of suitable habitats for all grid points when adding or subtracting physiological characteristic factors, and further compared the areas of suitable habitats lost or gained by the two relative higher or lower population groups divided at an elevation of 4200 m.

## 3 RESULTS

### 3.1 Measurement data

Active temperature, critical thermal maximum, and critical thermal minimum (mean ± SD) of four populations are shown in Table 2.

**TABLE 2:** Active temperature, critical thermal maximum, and critical thermal minimum (mean ± SD) of four populations.

	Active temperature (°C)	*n*	Critical thermal maximum (°C)	*n*	Critical thermal minimum (°C)	*n*
Zhongba	28.7 ± 6.31	34	41.19 ± 2.34	15	8.12 ± 1.76	15
Gar	28.1 ± 3.24	41	42.23 ± 2.47	15	10.01 ± 2.78	15
Dingjie	34.3 ± 5.0	15	45.64 ± 1.19	15	7.45 ± 1.59	15
Shigatse	34.3 ± 4.7	15	44.1 ± 1.53	15	8.09 ± 2.21	15

### 3.2 Data analysis

**FIGURE 2:**
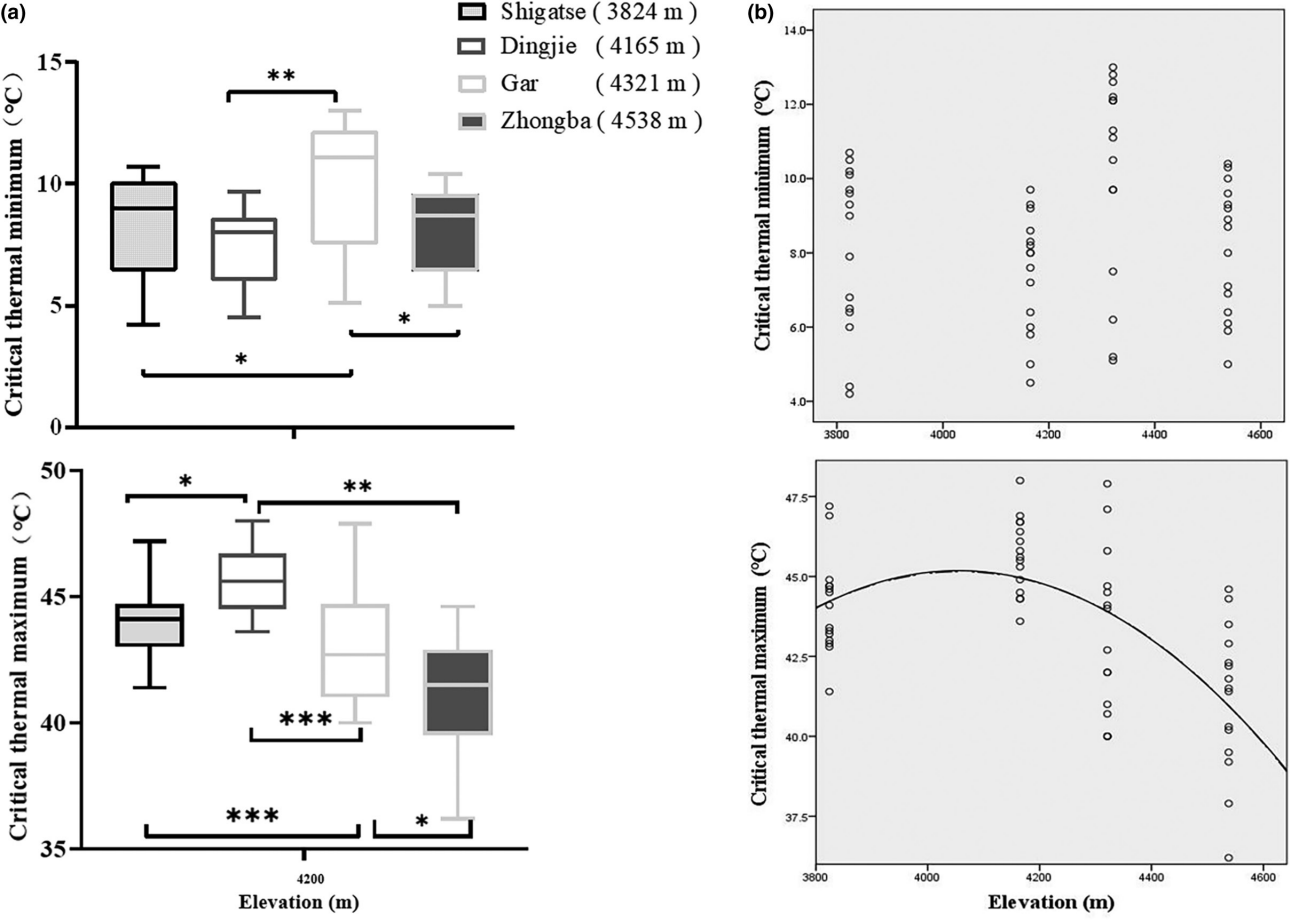
Significant differences in thermal tolerance and correlation relationships between CT_min_, CT_max_, and altitude among Zhongba, Dingjie, Gar, and Shigatse populations. (a) Paired differentiation of CT_min_ and CT_max_; (b) scatter plot of CT_min_ with altitude and the best fitting curve (quadratic model) for CT_max_ with altitude.

Tolerance temperature data for males and females within the same population were tested for normality using the Shapiro–Wilk test and for homogeneity of variance using Levene's test. The results indicated normality in all cases. There were no significant differences between males and females within the same population (*F*_2,58_ = 1.39, *p =* .36). After confirming the homogeneity of variances assumption (*p* > .05), we conducted comprehensive ANOVA tests for both CT_min_ and CT_max_. The overall ANOVA test yielded significant results (*F*_2,58_ = 13.01, *p =* .01), and similarly, for CTmin, the results were significant as well (*F*_3,57_ = 4.24, *p =* .01). These outcomes indicated that there was at least one sample significantly differed from the others. To further pinpoint the exact differences between specific population pairs, we employed the Tukey HSD (Honestly Significant Difference) test for post‐hoc contrasts. There was a lack of significant population paired differentiation of the observed CT_min_, except for the paired difference between Gar and the other three populations (Gar‐Shigatse: *F*_2,58_ = 4.23, *p =* .01; Gar‐Dingjie: *F*_3,57_ = 4.6, *p =* .001; Gar‐Zhongba: *F*_2,58_ = 4.30, *p =* .015), while the CT_max_ variable displayed a more frequent difference between populations (Shigatse‐Dingjie: *F*_2,58_ = 13.0, *p =* .01; Shigatse‐Gar: *F*_2,58_ = 13.1, *p =* .000; Dingjie‐Zhongba: *F*_3,57_ = 4.6, *p =* .000, Gar‐Dingjie: *F*_3,57_ = 13.6, *p =* .001; Gar‐Zhongba: *F*_2,58_ = 13.0, *p =* .011), as shown in Figure 2a.

The scatterplot indicated a significant correlation between CT_max_ and elevation (Spearman *r* = −.72, *n* = 15, *p* = .01) (Figure 2b). In contrast, the correlation between CT_min_ and elevation was not significant (Spearman *r* = .14, *n* = 15, *p* = .44). To delve deeper into the specific nature of the relationship between CT_max_ and elevation, we conducted a comprehensive model fitting analysis, using a linear model and 11 nonlinear models. We employed the Akaike Information Criterion to penalize more complex models, with the model achieving the smallest AIC value being identified as providing the best fit (Angilletta, 2006). The analysis revealed that CT_max_ showed a nonlinear relationship with elevation, with the quadratic models emerging as the most appropriate fit (*F*_2,58_ = 16.37, *R* ^2^ = .366, *p* < .05), which had the lowest AIC value of −41.96.

To assess the potential impact of using uniform shadow parameters across different altitudes, we calculated the mean vegetation shadow coverage rates for each site: Shigatse, Dingjie, and Zhongba, which were 0.16, 0.08, and 0.28, respectively. These figures revealed that the highest coverage is found at the highest altitude in Zhongba, but that the lowest altitude site, Shigatse, does not exhibit the lowest coverage. Our findings revealed no significant correlation between these two variables (Spearman *r* = −.93, *n* = 3, *p* = .71), with minimal variation in the average vegetation shadow coverage among different altitude samples.

To further explore the relationships between these factors, we controlled for both altitude and vegetation shadow coverage as variables and conducted Spearman correlation analyses on altitude versus activity time and vegetation shadow coverage versus activity time. Despite there only being three sites, the results indicated a perfect rank correlation between altitude and activity time (Spearman *r* = −.98, *n* = 3, *p* = .01), whereas no rank correlation was found between vegetation shadow coverage and activity time (Spearman *r* = −.50, *n* = 3, *p* = .67).

### 3.3 Suitable areas and shifts at different elevations

**FIGURE 3:**
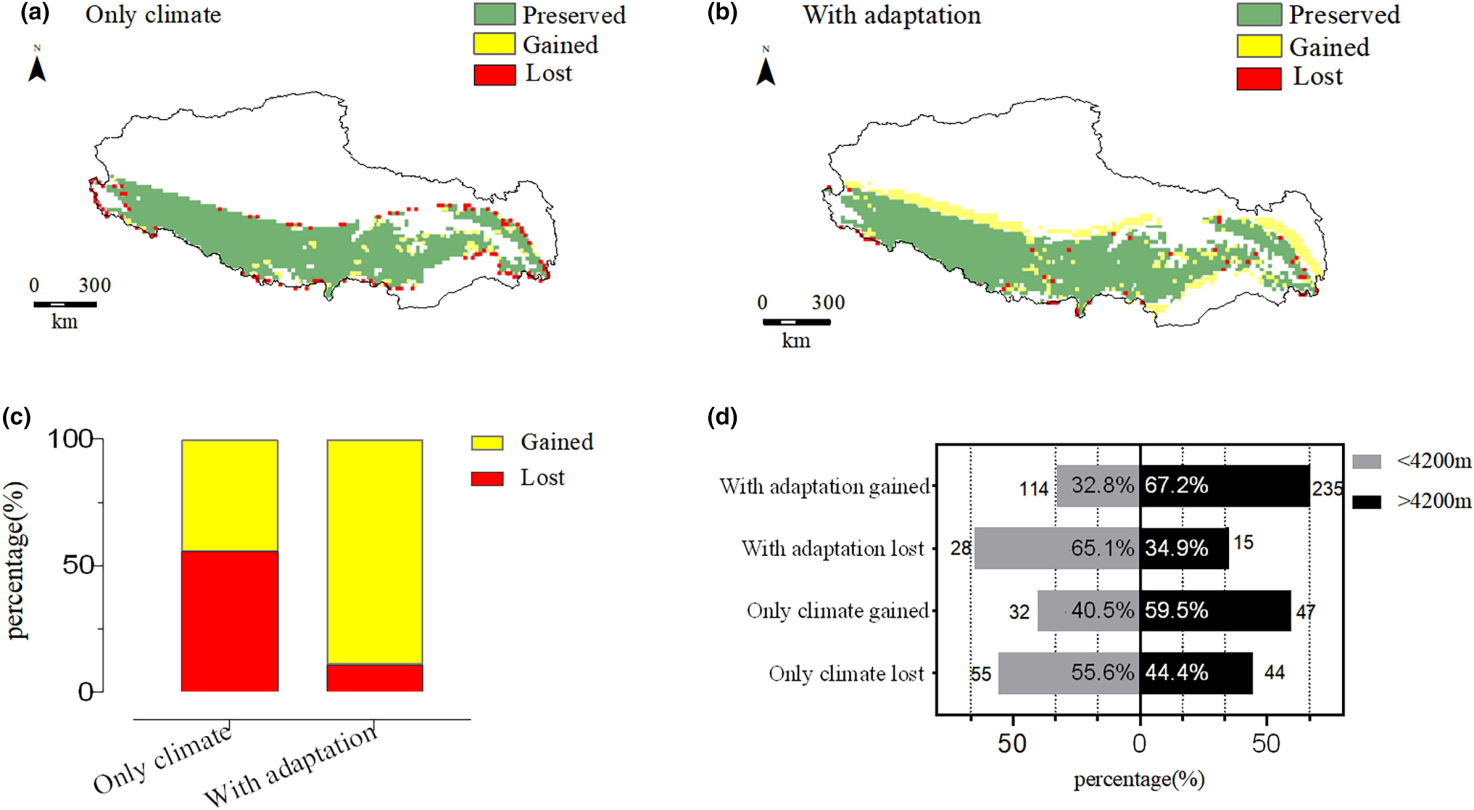
The impact of future climate scenarios on suitable habitats for *P. theobaldi*. The habitat mapping uses colors to illustrate changes: Green for areas of preserved habitable areas, yellow for gained areas, and red for lost areas. (a) Derived from climatic data only; (b) Includes physiological factors; (c) Compares the overall changes in suitable habitat area between the two scenarios; (d) A comparative bar graph illustrating the gained and lost potentially habitable areas at altitudes above and below 4200 m, with the numbers on top of gray and black bars indicating the count of grid cells for lost and gained suitable habitat (average grid cell size approximately 366 km^2^), and percentages at the bottom indicating the corresponding area proportions.

As anticipated, regions with suitable habitat exhibit alterations subsequent to a temperature rise. However, this effect is contingent on the inclusion of physiological factors in the simulation (Figure 3a,b). There is a decrease in the area of suitable habitat when climate alone is modeled, but suitable habitat is increased when physiological factors are also taken into account (Figure 3c). Furthermore, we examined the changes in area across different elevations using 4200 m as a threshold. Our study has revealed that, for *P. theobaldi* populations at elevations above 4200 m, when only climate variables are considered, the loss in habitable area represents 44.4% of the total decreased area, which is less than the loss experienced by populations living below 4200 m, where the loss constitutes 55.6%. However, when both climate and physiological factors are taken into account, the populations at elevations above 4200 m undergo an increase in habitable area that accounts for 67.2% of the total increased area, which is more than the increase for populations living below 4200 m, which accounts for only 32.8% of the total increased area. It has been observed that populations at higher elevations have either gained more suitable habitat or lost less compared to those at lower elevations when incorporating both climate and physiological factors into the analysis (Figure 3d).

### 3.4 Maximum activity time in the current climate

**FIGURE 4:**
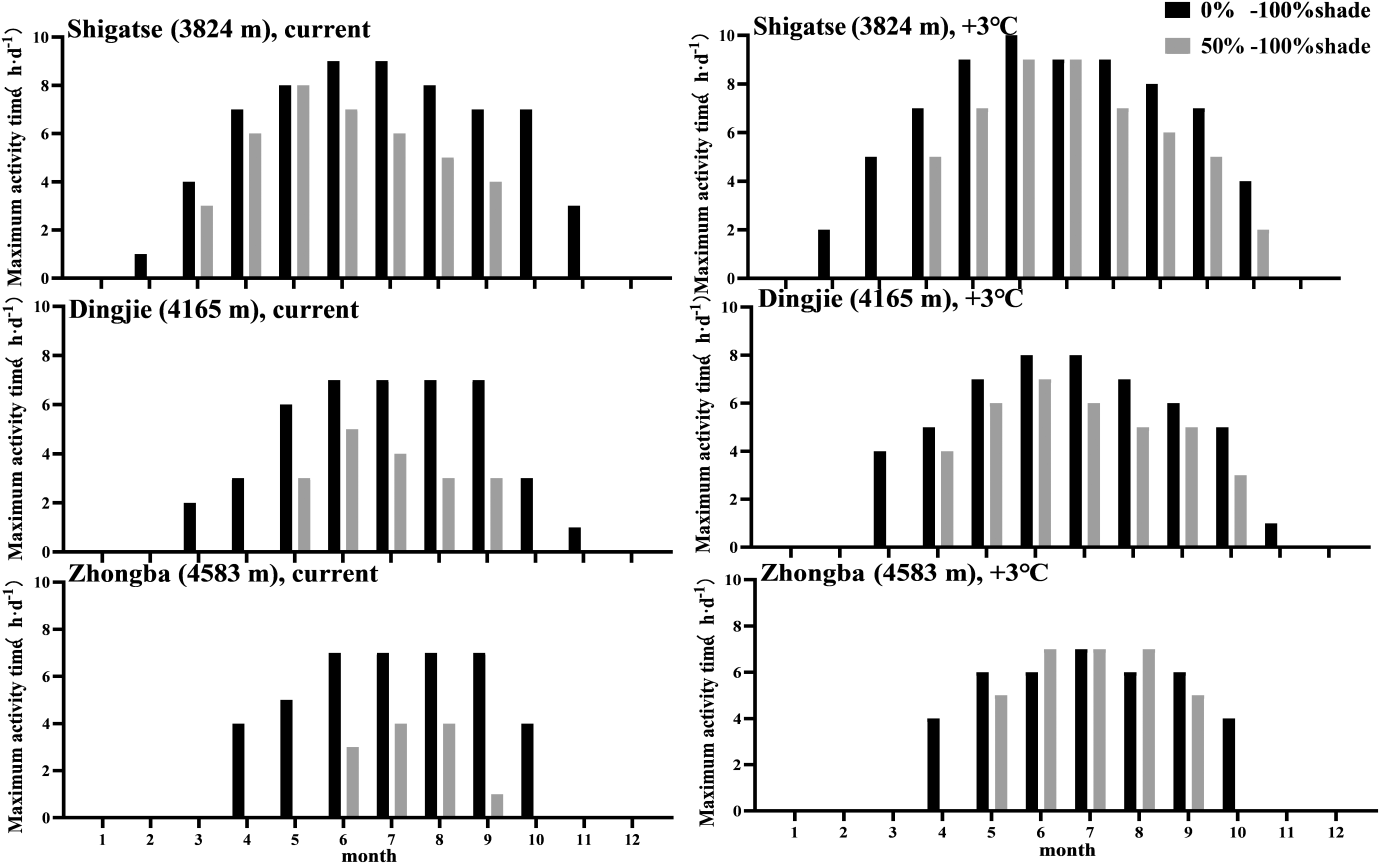
Bars indicate the maximum activity times at three different altitudes at Zhongba, Dingjie and Shigatse under the current climatic conditions (left column) and a 3°C temperature increase (right column). The number in parentheses is the altitude of these localities.

Under current climate conditions, the maximal activity time for the sand lizard is longer at lower elevations, decreasing with increasing elevation and more shade (Figure 4). During the warmest months (June to September), all three elevational populations of the *P. theobaldi* are predicted to exhibit their longest daily activity periods (7–10 h day^−1^, Figure 4). However, activity at the lowest elevation is predicted to span a greater part of the year and cover a longer part of the day during the coolest months (i.e., November, February to April, 4–7 h day^−1^, Figure 4) compared with the two higher elevations. The estimated total activity times at altitudes of 4583 and 4165 m are only 67% and 64% of the low elevation site, respectively (Table 3). Assuming minimal shading impact (0%–100% shading), the maximal activity time at mid‐elevation (1290 h year^−1^) is projected at 67% of the low elevation level (Shigatse, 1920 h year^−1^, Table 3). Accompanied by increased shading levels (50%–100% shading), there is a diminution in activity period (Figure 4, Table 3).

**TABLE 3:** The annual maximum activity time of three sampled population sites under different shading conditions. The numbers within the parentheses represent the percentage that this indicated maximum activity time constitutes of the maximum activity time under the current climate conditions (specifically, the 0%–100% shading levels observed in Shigatse).

Shade level	Shigatse (3824 m)	Dingjie (4165 m)	Zhongba (4583 m)
1. Current climate			
0%–100%	1920 h (100%)	1290 h (67%)	1230 h (64%)
50%–100%	1170 h (60%)	540 h (28%)	360 h (19%)
2. A 3°C increase			
0%–100%	2100 h (109%)	1530 h (79%)	1470 h (77%)
50%–100%	1500 h (78%)	1050 h (54%)	930 h (48%)

### 3.5 +3°C maximum active time

Increased maximum activity time is anticipated under future climate conditions with a 3°C rise in temperature (Figure 4). At Shigatse (low elevation), characterized by high levels of shade (50%–100%), lizards experience an extended period of peak activity, particularly from June to August, ranging from 2 to 6 h per day (Figure 4). At the same site, when characterized by low levels of shade (0%–100%), lizards display greater activity durations—approximately 109% of the duration observed in the current climate there (Table 3). Additionally, at high shade levels (50%–100%), there is a noticeable increase in activity duration compared to current climatic conditions. At the same time, mid and high elevations experience a more significant increase in activity time following the temperature rise (Figure 4; Table 3).

## 4 DISCUSSION

This study incorporated physiological characteristics such as CT_max_, CT_min_, and T_sel_ in model predictions and predicted a significant increase in the potential activity time and habitat range of the *P. theobaldi* populations by the end of this century, notably in high‐altitude areas. However, when considering only the warming climate variables in climate change scenarios, the overall suitable habitat of *P. theobaldi* is reduced, with a more pronounced decline at lower altitudes. Our results indicated that the physiological buffer of high altitudinal lizards should mitigate the effects of global warming.

Ectotherms at higher altitudes typically exhibit lower CT_max_ to adapt to extreme cold environments. While many studies suggest a significant decrease in CT_min_ with increased altitude, as observed in an island gecko and some Australian lizards (Brown, 1996; Senior et al., 2019), grasshoppers (Slatyer et al., 2020), and frogs (Gutierrez‐Pesquera et al., 2022), they report no substantial change in CT_max_ with altitude. However, analyses of interspecific patterns indicate a slight decrease in CT_max_ at higher elevations (Senior et al., 2019). Our research on the *P. theobaldi* reveals that populations at the highest altitudes have a lower CT_max_, with a distribution pattern that first increases then decreases, forming a nonlinear pattern in relation to altitude. These patterns suggest that lizards are largely capable of acclimating to the cooler temperatures at higher altitudes (Anderson et al., 2022; Brown, 1996), or alternatively, they could reflect the differentiation in thermal tolerance among populations at varying altitudes (Aubret & Shine, 2010; Caldwell et al., 2017).

Other studies also have identified a negative correlation between SVL and CT_max_ (Claunch et al., 2021; Wendt & Verble‐Pearson, 2016). Our previous work found a U‐shaped relationship between the SVL of the *P. theobaldi* and altitude (Jin & Liao, 2015). In conjunction with the observation of a nonlinear relationship between CT_max_ and altitude in *P. theobaldi*, our analysis further explored the correlation between SVL and body weight with CT_max_ either CT_min_ across specimens from the Gar and Zhongba regions. The results indicate that there is no significant correlation between SVL or weight and either CT_max_ or CT_min_. Consequently, we find no statistical evidence to suggest that SVL influences the variability of CT_max_. It is important to note that even if a significant correlation had been found, this would not necessarily imply a causal influence of SVL on CT_max_. Future research may focus on exploring other factors that could influence CT_max_ variability or employing longitudinal data to investigate potential causal relationships (see Table S2 for more details).

Climate change is predicted to contribute to a reduction in suitable habitat for *P. theobaldi* by the end of this century due to climatic factors alone, with more pronounced effects on populations below 4200 m in elevation. However, considering our physiological assessments, suitable habitat will not decrease in a manner that is consistent with the activity period patterns across various altitudes as described in this article, with habitats at higher elevations experiencing more significant growth. This is consistent with similar observations made for *P. vlangalii* (Jiang et al., 2023). As with other predictions for latitudinal variation analysis (Liu et al., 2022), our study reveals that the survival of the studied species may be threatened by climate change. However, when considering multiple climate variables and environmental changes, like some birds (Maggini et al., 2011) and insects (Moore et al., 2023), the lizards at higher altitudes are better adapted to counteract the negative effects of climate change due to their greater maximal activity time and the potential for habitable range expansion, compared to populations at relative lower altitudes.

Our study indicates that high‐altitude lizards are more active, supporting the notion that such populations have potential longer activity periods (Jiang et al., 2023; Jin et al., 2016; Van Damme et al., 1989; Yang et al., 2015). This enhanced activity may enhance feeding opportunities (Kearney et al., 2021) and survival adaptability (Kearney, 2013); particularly, populations at higher altitudes benefit from an abundance of potential prey (Lu et al., 2018). Shade also affects activity levels, with peak activity diminishing under increased shade. However, even with 50%–100% shade, the activity duration for populations at higher altitudes has similarly experienced a substantial increase. Consequently, *P. theobaldi* at high altitudes might gain an adaptive advantage due to climate change, possibly extending their range upward, which could prompt interspecific competition (Chen et al., 2011; Kolbe et al., 2010).

Given the relatively small variation in vegetation shadow coverage across the three altitudes and the absence of a significant correlation with activity time, it is plausible that the actual vegetation shadow coverage at these altitudes may not differ substantially from the results simulated using the shadow coverage intervals. However, the increase or decrease in altitude undoubtedly has a significant impact on activity periods.

We compared our predictive outcomes with video observation data collected from *P. theobaldi* population at the mid‐altitude Dingjie site. The videos meticulously documented the activity times of six individuals in August. The onset of their activities ranged from 10:23 to 11:09, while the cessation occurred between 16:04 and 17:00. Remarkably, the observed activity duration closely aligns with our simulated estimates, both indicating that the primary activity window spans from 10:00 AM to 5:00 PM.

Besides temperature, various biotic and abiotic factors can influence species distribution (Huang et al., 2020). Our focus is on how thermal adaptative and climatic variables affect the distribution of the lizard species. With a warming climate, the species is expected to benefit from increased activity times and a potential for range expansion. However, considering solely thermal factors, the influence may be adverse; ectotherms may employ various behavioral and physiological strategies to optimize energy use in response to elevated ambient temperatures (Dillon et al., 2010). While phenotypic plasticity in thermal adaptation can facilitate improved fitness, sustained high temperatures could overwhelm this plasticity, posing a risk of overheating (Gunderson et al., 2017; Seebacher et al., 2015), potentially reversing lizard fitness. Our findings contribute to a better understanding of lizard strategies in response to climate change and physiological adaptation, aiding in the conservation of their diversity.

## AUTHOR CONTRIBUTIONS

**Jie Gao:** Validation (equal); writing – original draft (equal). **Zian Wei:** Resources (equal). **Yuanting Jin:** Funding acquisition (equal); writing – review and editing (equal).

## FUNDING INFORMATION

This study was supported by the National Natural Science Foundation of China (Grant No. 32370441) and the Second Tibetan Plateau Scientific Expedition and Research Program (STEP, Grant No. 2019QZKK05010215).

## CONFLICT OF INTEREST STATEMENT

The authors declare no competing interests.

## Supporting information


Table S1.

Table S2.

Table S3.

## Data Availability

All data used in the article have been uploaded as supplementary files in the Tables [Link], [Link].
